# Effect of Dance Labor on the Management of Active Phase Labor Pain & Clients’ Satisfaction: A Randomized Controlled Trial Study

**DOI:** 10.5539/gjhs.v6n3p219

**Published:** 2014-03-30

**Authors:** Somayeh Abdolahian, Fatemeh Ghavi, Sareh Abdollahifard, Fatemeh Sheikhan

**Affiliations:** 1 Department of Nursing and Midwifery, Firuzabad Branch, Islamic Azad University, Fars, Iran; 2 Department of Nursing and Midwifery, Jahrom University of Medical Sciences, Jahrom, Iran; 3 Department of midwifery, Khalkhal branch, Islamic Azad University, Khalkhal, Iran

**Keywords:** dance, labor, management, pain, satisfaction

## Abstract

**Background:**

There are a wide variety of non- pharmacologic pain relief techniques for labor which include pelvic movement, upright position, back massage and partner support during the first stage of labor. The effectiveness of dance labor- which is a combination of these techniques- has not been evaluated.

**Aim:**

This study aimed to evaluate the effectiveness of dance labor in pain reduction and woman’s satisfaction during the first stage of labor.

**Methods:**

60 primiparous women aged 18-35 years old were randomly assigned to dance labor and control groups. In the dance labor group, women were instructed to do standing upright with pelvic tilt and rock their hips back and forth or around in a circle while their partner massaged their back and sacrum for a minimum of 30 minutes. In the control group, the participants received usual care during physiologic labor. Pain and satisfaction scores were measured by Visual Analogue Scale. Data were analyzed by using the t. test and Chi-square.

**Findings:**

Mean pain score in the dance labor group was significantly lower than the control group (P < 0.05). The mean satisfaction score in the dance labor group was significantly higher than in the control group (P < 0.05).

**Conclusion:**

Dance labor which is a complementary treatment with low risk can reduce the intensity of pain and increase mothers, satisfaction with care during the active phase of labor.

## 1. Introduction

Women are increasingly expecting to participate in decisions about their healthcare, especially during childbirth (Lothian, 2009). There are choices to be made during childbirth such as labor pain relief methods, and each method has risks and benefits, with different effectiveness, availability, and acceptability (Lally, Murtagh, Macphail, & Thomson, 2008). There are various kinds of non-pharmacologic pain relief techniques which include positioning, movement, and massage (Simkin & Bolding, 2004).

One common non-pharmacologic method is the upright position during the first stage of labor (Storton, 2007). The supine position is purported to adversely affect heart rate and blood flow of the fetus and might increase maternal stress hormones, thereby decreasing uterine contractility and progress of labor (Lawrence, Lewis, Hofmeyr, & Dowswell, 2009). But the upright position during the first stage of labor uses gravity to help contractions, while decreasing the pain most women feel (Souza, Miquelutti, Cecatti, & Makuch, 2006) and this may improve maternal comfort and reduce the need for analgesia (Simkin & O’hara, 2002). In addition, labor without bed confinement became a symbol ofmothers, empowerment and the humanization of labor (Souza et al.,2006). Also the upright position enhances the descent of the fetal head with a shorter duration in first and second stages of labor (Liu, 1989; Calvo Aguilar, Flores Romero, & Morales, 2013; Lawrence, Lewis, Hofmeyr, Dowswell, & Styles, 2009).

The upright position can be help ful until the mother has enough energy to be upright; and then leaning on a labor partner makes it easier for the mother to support her body weight (http://www.birthingnaturally.net/cn/position/dance.html). Support by a family member during delivery could significantly decrease the number of invasive procedures during and after the delivery (Kaźmierczak, Fiegler, Wegrzyn, & Cholewa, 2006). This finding has strong implications for maternity practices in countries such as Islamic Republic of Iran, in which maternity wards rarely encourage husbands be present during childbirth (Sapkota, Kobayashi, Kakehashi, Baral, & Yoshida, 2012). The World Health Organization (WHO) (2009) has recommended that a parturient woman be allowed to have a birth companion she trusts and with whom she feels at ease. However, these recommendations do not tend to be followed in facility-based births in many developing countries, including Islamic Republic of Iran.

In addition, the massaged mothers, either on the back or sacrum in the first stage or on perinea in the second stage, reported a decrease in pain and also had significantly shorter labors, shorter hospital stay and less postpartum depression (Field, Hernandez-Reif, Taylor, Quintino, & Burman, 1979; Hajiamini, Masoud, Ebadi, Mahboubh, & Matin, 2012; Sanders, Peters, & Campbell, 2005)

Pelvic tilt exercise appears to be effective in reducing ligament pain intensity and also pain duration. As a nurse-midwifery strategy, this exercise promotes patient comfort and facilitates self-care in pain relief during pregnancy (Andrews & O’Neill, 1994). Pelvic movement or rocking, either on a chair or swaying back and forth, allows the woman’s pelvic to move and encourages the fetus to descend. It must be reinforced that in upright position, gravity helps delivery of the fetus (Lawrence et al.,2009).

These inexpensive non-pharmacologic methods can be combined or used sequentially to enhance the overall effect (Simkin & Bolding, 2004). These combinations of upright position, pelvic movement, back massage, and partner support during the first stage of labor has been termed dance labor. The dance labor with music to encourage a gentle rhythm promotes a very relaxing environment and allows the partner to have access to the mother’s back for massage or pressure (Simkin & Ancheta, 2011).

In Iranian society, vaginal birth is anticipated as a painful and lengthy process, with low cultural acceptance and resulting in less income for obstetricians. Therefore qualitative study conducted in Islamic Republic of Iran showed that most of the factors identified by participants facilitated the choice of cesarean section (Bagheri, MasoudiAlavi, & Abbaszadeh, 2013). Currently there are few educational opportunities and limited researches on complementary and alternative medicine used in midwifery practice; these shortfalls need to be addressed by the profession (Halla, McKennab, & Griffiths, 2012).

Women may have ideal hopes of what they would like to happen with respect to pain relief, control, and engagement in decision-making, but experience is often very different from expectations (Lally et al.,2008). The massaged mothers reported a decrease in depressed mood and anxiety, showed less agitated activity and had more positive affect during labor (Field et al., 1997). Also, when a woman’s husband is present at birth, she feels more in control during labor (Sapkota et al.,2012) and this helps to reduce maternal anxiety during childbirth (Chunuan et al.,2009) and finally leads to a more positive birth experience (Campero et al.,1998). In the literature there is no study about the effects of dance labor on pain relief and satisfaction of women.

The purpose of this study was to evaluate the effectiveness of dance labor in pain relief and the woman’s satisfaction during the first stage of labor.

## 2. Subjects and Methods

In this randomized controlled trial using convenience sampling, 60 volunteer primiparous women were recruited from one of the large general public hospitals of Shiraz University of Medical Sciences, in Fars province- Iran. The study protocol was approved by the ethics committee of Shiraz University of Medical Sciences, and ethical permission was obtained from this committee.

Demographic characteristics such as age, education level, gestational age, and occupation were obtained from the medical records of participants. The investigator, who was an experienced midwife, performed a clinical examination to record dilatation, effacement, station, position, duration and interval of uterine contractions, and fetal heart rate.

The study sample included primiparous women aged 18 to 35 years old with single pregnancies, cephalic presentation of fetuses, 38 to 40 complete weeks of gestation, anticipation of a normal birth, and no history of infertility. After describing the aim of the research and obtaining informed consent, we randomized those women in the first stage of active-phase labor with cervical dilatation between 4 and 10 centimeters into 2 groups. Randomization was accomplished with a table of random numbers. If the number was even, the woman was assigned to the dance labor group (group 1), and if the number was odd, the woman was assigned to the control group (group 2). If there was a need for analgesic medication, or if obstetric complications occurred, the participant was immediately referred to an obstetrician and other professionals as needed, then excluded from the study.

In the dance labor group, women were instructed to do standing upright with pelvic tilt and rock their hips back and forth or around in a circle while their partner-who was instructed to stand in front of them, massaged their back and sacrum for a minimum of 30 minutes. During these movements, participants were instructed to rest their arms on their partner’s shoulders. Women in this group were instructed to remain upright at least for 30 minutes to record pain score.

In the control group, the participants could select their own position and received usual care during physiologic labor, without ambulating or any intervention.

In both groups all stages of labor were completed in a labor room with equal environmental conditions such as room temperature, light, sound, equipment. No pain management intervention was provided to the control group. Women in both groups spent active phase of labor with their husband or family members and usual clinical examination (station, dilatation, effacement) was accomplished every 2 hours, and fetal heart rate was monitored every 30 minutes throughout the active phase of labor.

The study was supervised by an experienced midwife and in both groups the pain score was recorded by the participants using a visual analogue scale (VAS) of 0 (lack of pain) to 10 (most severe pain they had experienced). Pain scores were measured in both groups before labor and then obtained every 30 minutes in both groups until cervical dilation reached 10cm.

VAS was used for measurement of satisfaction as well as the pain recordings (0-10 cm, 0=worst possible, 10=best possible). The measurements of satisfaction were accomplished after birth and the mothers in both groups were asked to score their satisfaction about birth process.

To further reduce bias, researchers were instructed not to give verbal information about the possible effects of the dance labor to participants before and during the study. Also, the individual responsible for data analysis was masked to the study purposes to minimize any bias that might arise from knowledge about the participants. This ensured us that, as far as possible, differences came only from the effect of dance labor usage.

In this study the pain scores, duration of the active phase and satisfaction in dance labor and control groups were compared by using the t test in SPSS version 14. The demographic characteristics were analyzed by t test and chi-square test. P value less than 0.05 was considered significant.

## 3. Results

Sixty primiparous women were enrolled in this study. Demographic characteristics of subjects (mean age, educational level, occupation and gestational age) are shown in Table 1.

**Table 1:** Demographic characteristics of subjects in control and dance labor groups

Characteristic	Control	Dance labor	P. Value
**Mean age (Mean ± SD)**	25.13±4.82	22.96±4.37	0.07
**Educational Level (Under diploma)**	36.66%	40%	0.062
**Occupation (House keeper)**	96.66%	86.66	0.23

The mean score of pain severity in the dance labor group was significantly less than that of the control group.

**Figure 1:**
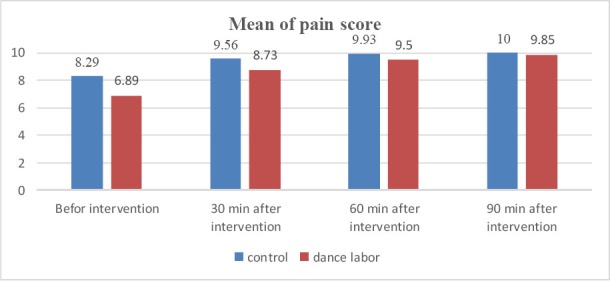
Mean of pain score in dance labor and control groups evaluated by (VAS)

There were significant differences between the pain scores of the women in the dance labor group before intervention (p=0,008) and 30 min after intervention (p=0.012) and 60 minutes after intervention (p=0.036) when compared with the pain scores of the women in the control group (Figure 1).

Also, this study showed no significant difference in the duration of active phase of labor between groups.

There was significant difference in the mean scores of satisfaction between the two groups (p=0.021). The mean satisfaction score in the dance labor group was significantly higher than control group. (Table 2)

**Table 2:** Mean of satisfaction score after birth in dance labor and control groups evaluated by (VAS)

Satisfaction	Control	Dance labor
**Mean±SD**	4.13 ±1.041	4.66±0.6609
**P.Value**	P= 0.021*	

* Significant.

## 4. Discussion

This study found that dance labor can reduce the intensity of pain and increase the satisfaction of mothers during the active phase of labor. However, no study evaluated effects of dance labor but the effects of massage therapy, upright position, and partner contribution on pain labor, duration of active phase of labor, and maternal satisfaction have evaluated (Simkin & Bolding, 2004; Tournaire & Theau-Yonneau, 2007).

In our study, duration of first stage of labor was not significant between groups. Therefore upright position as a safe and well-accepted option for the women during the first stage of labor might not contribute towards a shorter duration of labor. But Lawrence’s study showed that first stage of labor for upright women was approximately one hour shorter than recumbent women (Lawrence et al.,2009). In the study of Andrews and O’Neill (1994) women in the upright position group had significantly shorter phase of maximum slope in labor). In our study, labor pain was measured during active labor phase, hence not permitting the determination of the effect of dance labor during different stages of labor. Lawrence’s (2009) study showed that there were no differences between upright women and recumbent women groups for length of the second stage of labor, or wellbeing of women and babies. The systematic review of six trials showed that three trials showed decreased pain in upright positions, two found no difference, and one (in which women were forced to remain upright throughout the first stage) found increased pain (Gupta & Hofmeyr, 2012). In our study, the upright women were forced to remain upright at least for 30 min to record pain and they had significantly lower pain than control group. Since standing position and pelvic movement cause women to become tired, most women in intervention group wanted to lie down after 90 min. Liu found that upright position enhanced the descent of the fetal head with a shorter duration of labor in first and second stages (Liu, 1989).

Effect of dance labor on pain was consistent with the findings of other studies which showed that there was significant reduction in pain reported by women in the massage group (Sanders et al.,2005), upright position (Simkin & O’hara., 2002) pelvic tilt (Suputtitada, Wacharapreechanont, & Chaisayan, 2002) accompanied by husband (Sapkota et al.,2012) or family at birth (Susan, Lindner, Jacqueline, & McGrath, 2012). This is also in agreement with findings by Janssen, Shroff and Jaspar (2012) which demonstrated that massage in the first stage of labor reduced pain severity in pregnant women.

In our study, pain score in the dance labor group was significantly lower than the control group but Janssen et al. reported that scores on the McGill Pain Scale were insignificantly lower in the massage group. It must be stated that in our study back massage by the partner was combined with pelvic tilt and upright position was applied during the first stage of labor, but in the study by Janssen et al. (2012), massage was applied by a massage therapist on different locations of body. In the study by Field et al. (1997) massage of head, back, hands and feet of pregnant women by their partners during labor caused less pain and anxiety in women, and improved their mood. In the study by Chang et al. massage was performed three times and pain intensity, rated by using a present behavioral intensity (PBI) scale, reduced in the massage group at each phase of labor (Chang, Wang, & Chen, 2002). In another study performed by Chang et al. massage reduced pain intensity at cervical dilation up to 7 cm, but after this phase there were no significant differences between the groups. However, the study design and duration time of massage (60 min) was different from other studies (Chang, Chen, & Huang, 2006). Since in our study the participants in the control group only received usual care during labor, more attention to the intervention group by their partners might affect reporting pain scores and this might cause bias in the findings.

In this study dance labor reduced the pain scores reported by women during the active phase of labor. In the study of Taavoni et al. (2011) pelvic tilt by using a birth ball had no effect on the duration of the active phase of labor but this complementary treatment could reduce the pain during labor.

In other studies, sitting pelvic tilt exercises during the third trimester in primi gravid reduced back pain intensity (Suputtitada et al., 2002; Susan et al., 2012). However it must be stated that pelvic floor muscle activation during vaginal delivery might represent an obstacle to fetal descent and increase the risk of pelvic floor injuries (Parente, Natal Jorge, Mascarenhas, & Silva-Filho, 2010). Dance labor might detract the attention of women during labor and therefore reduce pelvic floor muscle activation. Mothers who have the support of a partner during delivery experience fewer childbirth complications and less postpartum depression (Iliadou, 2012). Since family support might cause bias in results, in our study partners were present in both groups. Providing the information to mothers, partners, and family members allows the pregnant women to feel that she is not alone during labor (Susan et al., 2012). Birth is the beginning of fatherhood for men and their lack of knowledge causes their unclear role during labor (Longworth & Kingdon, 2011). In our study, the pain score was not different between women who had husband and those who had another family member as partner during labor.

In recent decades, the importance of satisfaction with health care has been emphasized (Kankaanpaa, Taimela, Airaksinen, & Hanninen, 1999) and this is being used by health care managers in evaluating the quality of care, and by policy makers in making decisions about the organization of health services (Flint, 1997).

One trial assessed satisfaction of walking option during labor, which was very high in the upright group. No trial found any harm associated with the upright position (Gupta et al.,2012).

There are different methods to evaluate satisfaction and VAS, although a crude measurement, is one of many well-recognized methods to measure satisfaction (Brown & Lumley, 1997).

In this study the mean satisfaction score in dance labor group was significantly higher than in the control group. It is in agreement with the findings of Zahrani et al. that showed the application of back massage during labor increased mothers, satisfaction (Zahrani, Honariou, Jannesari, & Alavimajd, 2006).

In the study of Andrews and O’Neill (1994), the comfort level of upright women was not significantly different from women in the recumbent group.

In our study, satisfaction of mothers about the total experience of childbirth was evaluated but it seems that if women be able to successfully manage their childbirth pain, they may evaluate themselves more satisfactorily than they evaluate the total experience. Therefore, measuring only total childbirth satisfaction may give an incomplete reflection of women’s satisfaction with the childbirth experience. It might be helpful in future studies if the comfort level of women during dance labor would be evaluated.

Many conceptualizations of satisfaction refer to expectations as a major determining factor of satisfaction (Hodnett, 2002). It must be stated that women whose expectations for childbirth were met are more satisfied than those whose expectations were not (Christiaens & Bracke, 2007). Expectations are related to several aspects of delivery, such as emotions (Goodman, Mackey, & Tavakoli, 2004), labor duration (Booth & Meltzoff, 1984), the need for interventions (Goodman et al.,2004), the condition of the child (Booth & Meltzoff, 1984) and the support of the partner and the medical staff (Gibbins & Thomson, 2001). The expectations of mothers were not considered in this study.

Limitations of our study should be considered. In this study, history of pain experience was not evaluated but this item could have effects on labor pain score. Although masking of women and their birth attendants was not possible, the person who analyzed the data was not informed about the aim of our study.

However future studies might be necessary to evaluate dance movements effectiveness during pregnancy and postpartum period. Dancing movements of women with their husbands during pregnancy might improve the relationship of family members and also these exercises might help them to do dance labor during labor.

## 5. Conclusion

Dance labor, which is a complementary treatment with low risk, can reduce the intensity of pain and increase the satisfaction of mothers with care during the active phase of labor.
